# A Novel Assessment of Sagittal Proximal Tibial Morphology and Relationship to Proximal Posterior Tibial Slope: Lateral Supratubercle Angle

**DOI:** 10.1177/03635465251331005

**Published:** 2025-04-15

**Authors:** Alfred Mansour, Alexis Aboulafia, Nicole Lemaster, Jessica Dziuba, Nikhil Gattu, Hayden Anz, William Brooks, Jaremy Rodriguez, Walter Lowe

**Affiliations:** † University of Texas Health Science Center at Houston, Department of Orthopaedic Surgery, McGovern Medical School, Houston, Texas, USA; ‡ Memorial Hermann Rockets Sports Medicine Institute, Houston, Texas, USA; Investigation performed at the University of Texas Health Science Center at Houston, Houston, Texas, USA

**Keywords:** knee, ACL, slope-reducing osteotomy, posterior tibial slope

## Abstract

**Background:**

Multiple techniques have been utilized to measure posterior tibial slope (PTS) without consensus on which imaging modality, view, and axis combination is most consistent for risk assessment and preoperative planning in primary and revision anterior cruciate ligament (ACL) surgery. An exclusively proximal-based measurement of PTS has yet to be defined.

**Purpose/Hypothesis:**

The purpose of this study was to establish normal values for novel measurements of sagittal proximal tibial morphology, the lateral supratubercle angle (LSTA) and the lateral supratubercle distance (LSTD), in normative and primary ACL tear cohorts. The secondary aim was to establish cutoff values and determine if these tibial measurement values are predictive of the presence of an ACL tear. It was hypothesized that LSTA will be significantly different between cohorts.

**Study Design:**

Case-control study; Level of evidence, 3.

**Methods:**

The medical records of patients with a knee complaint between August 2016 and June 2024 were retrospectively reviewed, and the patients were placed into either the normative or primary ACL tear cohort. Three independent observers measured LSTA, LSTD, and PTS along both the lateral (L) and medial (M) tibial plateaus on standard lateral knee radiographs. Means were calculated for each measurement and compared between groups. The receiver operating characteristic curve was used to determine the sensitivity and specificity of significant measurements.

**Results:**

Significant differences were found between normative (n = 150) and primary ACL tear (n = 150) groups in LSTA-L (normative: 9.9°± 4.4° vs primary ACL tear: 11.1°± 4.4°; *P* = .02), LSTA-M (normative: 10.3°± 4.4° vs ACL tear: 11.4°± 4.6°; *P* = .03), and PTS-M (normative: 9.2°± 3.2° vs primary ACL tear: 10.0°± 3.1°; *P* = .03).

**Conclusion:**

Mean values and ranges for LSTA and LSTD have been established in normative and primary ACL tear cohorts. LSTA-L, LSTA-M, and PTS-M significantly differed between the cohorts. Future studies with LSTA will evaluate the utilization of these proximal tibial deformity–based measurements in ACL surgery, retear risk assessment, and slope-reducing osteotomy planning.

The posterior tibial slope (PTS) has been used to quantify sagittal plane tibial deformity, which has been implicated in anterior cruciate ligament (ACL) rupture.^16,29,35,37,45,46^ PTS is defined as the angle between the longitudinal axis of the tibia and the posterior inclination of the tibial plateau.^6^ An increased PTS increases tibial shear force and anterior tibial translation, placing more stress on both native and reconstructed ACLs.^10,26,39^ Elevated PTS has been reported to increase the risk of ACL graft failure, with a PTS ≥12° being the suggested threshold.^3,24-26,32,38^

While PTS accounts for sagittal plane tibial deformity and measuring it has become increasingly common for patients with ACL injuries, there is variability in the best technique and modality for measuring it.^§^ Currently, there is a not a gold standard technique for measuring PTS, particularly regarding whether the anatomic or mechanical axis of the tibia provides the most accurate measurement.^5,15,17,48^ There is even less of a consensus on the best modality to measure PTS, including standard radiographs, full-length radiographs, magnetic resonance imaging (MRI) scans, and computed tomography (CT) scans.^5,12,19,27,30,34,44^ Some studies have reported more accuracy in PTS measurement on full-length radiographs over standard knee radiographs,^12,28^ while others have reported no difference between the 2.^5^ Such variability raises the question as to which technique and modality most consistently represent the sagittal deformity.

Slope-reducing osteotomy has been successfully used in revision ACL reconstruction cases to decrease anterior tibial translation and forces across the ACL graft.^7,21,41,45,47^ Deformity correction of elevated PTS occurs at several levels—supratubercle, transtubercle, and infratubercle—without a clear understanding of (1) the primary region of deformity, (2) which level of osteotomy addresses the primary region of deformity, and (3) the effect of performing deformity correction away from the center of deformity. This lack of clarity, in addition to the variability with measuring PTS, has led us to propose the measurement of a novel angle, lateral supratubercle angle (LSTA), and distance, lateral supratubercle distance (LSTD), along both the medial and lateral tibial plateaus to better understand the deformities in the region superior to the tibial tubercle.

As such, this study aimed to establish normal values for novel measurements of sagittal proximal tibial morphology, LSTA and LSTD, within a normative cohort and a primary ACL tear cohort. The secondary aim was to examine the relationship between these measurements and PTS on standard lateral knee radiographs. We hypothesized that LSTA would be significantly different between cohorts and provide a more specific location-based assessment of the supratubercle region deformity.

## Methods

This retrospective cohort study consisted of 2 disparate groups: the normative and primary ACL tear. Institutional review board approval was obtained at the senior author’s (W.L.) institution. This study reviewed the medical records of patients with a knee complaint seen by 1 of 2 surgeons (A.M., W.L.) between August 2016 and June 2024. An a priori power analysis was conducted using G*Power Version 3.1.9.7^13^ for sample size estimation based on our previously presented preliminary data (N = 100), which compared LSTA-M in a control group to LSTA-M in a primary ACL tear group.^14^ The effect size in this study was 0.40, which is considered to be small using Cohen criteria.^4^ With significance criteria of α = .05 and power = 0.90, the minimum sample size needed was 139 for each group. Therefore, this study screened patients until 150 were placed in the normative group and 150 in the primary ACL tear group.

To begin screening for eligible patients, International Classification of Diseases, 10th Revision (ICD-10) codes for ACL sprain or tear were extracted from an electronic health record software system for the primary ACL tear cohort between January 2018 and December 2023. The following ICD-10 codes were extracted for the normative cohort: recurrent patellar subluxation, recurrent patellar dislocation, patellofemoral disorders, other derangements of the patella, chondromalacia of the patella, other disorders of the patella, unspecified disorder of the patella, plica syndrome, prepatellar bursitis, other bursitis of the knee, osteochondritis dissecans, knee chondromalacia, contusion of the knee, open wound of the knee, and superficial injuries to the knee and lower leg.

**Figure 1.**
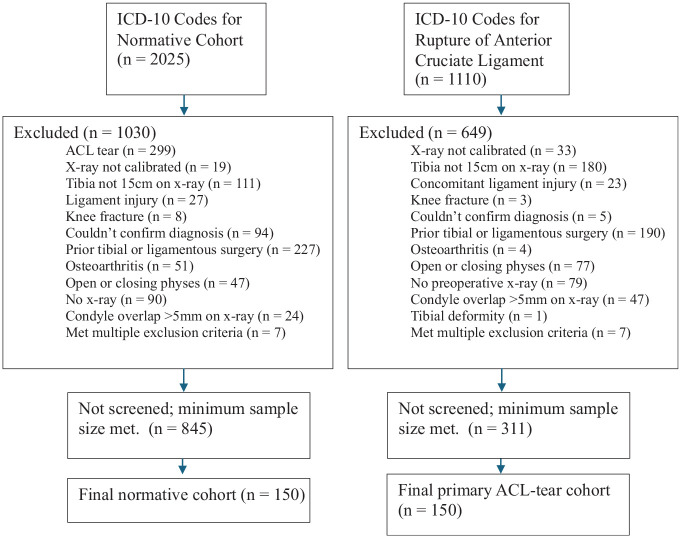
STROBE (Strengthening the Reporting of Observational Studies in Epidemiology) diagram depicting patient selection criteria. ACL, anterior cruciate ligament; ICD-10, International Classification of Diseases, 10th Revision.

Inclusion criteria for both cohorts included skeletal maturity, absence of arthritis on knee radiographs, previous tibial surgeries, and no lower extremity fractures at the time of imaging. Patients allocated to the primary ACL tear group were included once their ACL tear was confirmed by MRI. Patients were excluded from both cohorts if they had a history of ACL tear, had a previous ACL injury, and/or had repair/reconstruction on the ipsilateral knee. The control group included patients who underwent lateral standard knee radiographs for knee complaints unrelated to an anterior cruciate, posterior cruciate, medial collateral, or lateral collateral ligament or posterolateral corner injury (Figure 1).

Patients who met the inclusion criteria for either group underwent further screening of their standard lateral knee radiographs. Radiographic requirements included calibrated radiographs with a centered knee and a posterior femoral condyle obliquity <5 mm, as measured between the medial and lateral femoral condyles,^11^ and a minimal visible tibial length of 150 mm from the tibial plateau.

Three independent raters (2 medical students, 1 research coordinator) (A.A., J.D., N.G.) measured LSTA, LSTD, and PTS, utilizing both the medial (M) and lateral (L) plateaus as separate values, for a total of 6 measured values per radiograph. Each rater performed these measurements on 100 blinded radiographs. Raters were trained by a dual fellowship–trained orthopaedic surgeon and underwent reliability testing. The reliability data were extracted from a random selection of 15 patients in the normative cohort and 15 in the primary ACL tear cohort.

### Identifying the Medial and Lateral Tibial Plateaus

**Figure 2.**
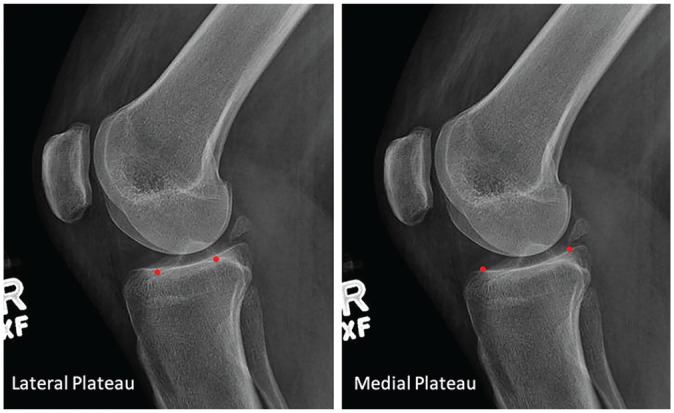
Identification of the anterior and posterior tibial plateau points (red dots) on both the lateral and medial plateaus.

The most anterior and posterior points of the sclerotic portion of each plateau were chosen (Figure 2). These points were utilized when drawing a line along each tibial plateau.

### Measuring LSTA

**Figure 3.**
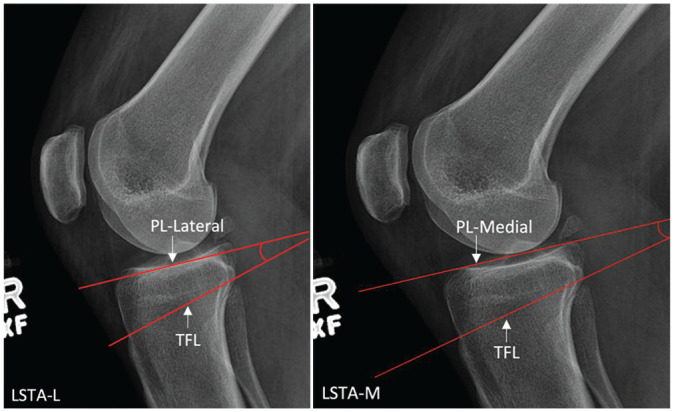
An example showing the lateral supratubercle angle medial (LSTA-M) and the lateral supratubercle angle lateral (LSTA-L). PL, plateau line; TFL, tubercle-flare line.

LSTA was defined as the angle formed between the line connecting the superior edge of the tibial tubercle to the proximal posterior cortical flare of the tibia (tubercle-flare line) and the line tangent to the anterior-most edge and posterior-most edge of the respective tibial plateau (plateau line [PL]) (Figure 3). Separate measurements were obtained for lateral and medial tibial plateaus (LSTA-L and LSTA-M).

### Measuring LSTD

**Figure 4.**
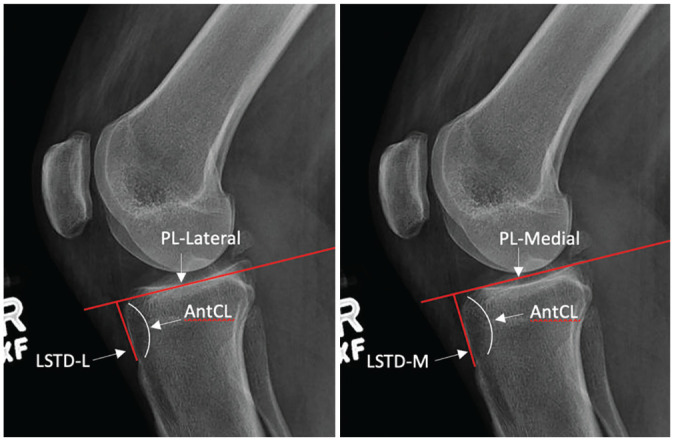
An example showing the lateral supratubercle distance medial (LSTD-M) and lateral supratubercle distance lateral (LSTD-L). AntCL, anterior cortical line; PL, plateau line; TFL, tubercle-flare line.

The LSTD was defined as the distance created by forming a line beginning at the superior edge of the tibial tubercle that follows the anterior cortex (anterior cortical line) until it meets a line tangent to the respective tibial plateau extending anteriorly on the standard lateral knee radiograph (PL) (Figure 4). Separate measurements were obtained for lateral and medial tibial plateaus (LSTD-L and LSTD-M).

### Measuring PTS

**Figure 5.**
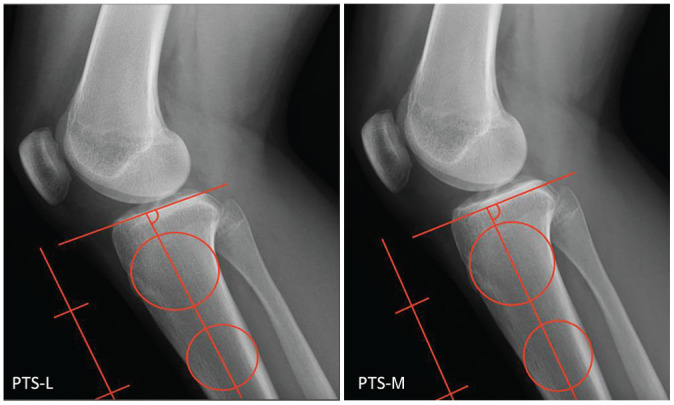
An example showing the posterior tibial slope (PTS) measurement technique utilized along the lateral, L, and medial, M, plateaus.

The PTS measurements were created by placing 2 best-fit circles in the proximal tibia centered at 50 mm and 150 mm, with each circle contacting the anterior and posterior cortices according to previously published techniques (Figure 5).^5,34^ A line from the tibial intramedullary canal was drawn bisecting both circles before intersecting with a line drawn tangent to the respective tibial plateau. Separate measurements were obtained for lateral and medial plateaus (PTS-L and PTS-M).

### Statistical Analysis

The mean values, ranges, and standard deviations for all 6 measurements (LSTA-L, LSTA-M, LSTD-L, LSTD-M, PTS-L, and PTS-M) were calculated for both the normative and primary ACL tear groups. Reported values included ranges of 1 standard deviation from the mean. Data were checked for normality using a Kolmogorov-Smirnov test for normality. Based on the overall means, differences between the normative and primary ACL tear groups were evaluated with a *P* value <.05 to indicate statistical significance using an independent *t* test. All data were analyzed using statistical package SPSS Version 28 (IBM Corp). Cohen *d* effect sizes were calculated with values between 0.2 and 0.49 considered small, between 0.50 and 0.79 moderate, and ≥0.80 large.^4^

To establish reliability of data collection for each measurement, interrater and intrarater intraclass correlation coefficient (ICC) values were utilized. ICC was measured using a 2-way random-effects model. The absolute measure of ICC agreement was used in all reliability analyses versus consistency measure. The consistency measure of ICC agreement, which treats any systematic bias between measurement groups as irrelevant, was not deemed appropriate.^18^ Intrarater ICC indicates the reliability of measurements within each observer. Interrater ICC indicates the similarity of measurements between observers. A *P* value <.05 was used to indicate absolute agreement within and between raters, including systematic errors and random residual errors.

A receiver operating characteristic (ROC) curve was created to determine the threshold of tibial measurements that demonstrated statistically significant differences between the normative and primary ACL tear groups. Mean values of the primary ACL tear cohort were used to establish cutoff values for all significant measurements. Sensitivity and specificity equivalent to the cutoff threshold were reported. The area under the curve (AUC) was calculated as a representative of value accuracy. An AUC <0.5 suggests no discrimination, 0.5 to 0.69 poor discrimination, 0.7 to 0.79 acceptable discrimination, 0.8 to 0.89 excellent discrimination, and 0.9 to 1.0 outstanding discrimination.^31^

## Results

In total, 300 patients, 150 in each cohort, were included (142 female, 158 male). The normative cohort consisted of 87 male and 63 female patients. The primary ACL tear cohort consisted of 71 male and 79 female patients. There was no significant difference in sex between the normative and primary ACL tear cohorts (*P* = .06). The total mean age at the time of radiograph was 26.41 ± 13.34 years (normative cohort: 32.58 ± 15.08 years; primary ACL tear cohort: 20.11 ± 7.53 years). There was a statistically significant difference in age between groups (*P* < .001). All data were normally distributed.

Means were calculated for LSTA-M, LSTA-L, LSTD-M, LSTD-L, PTS-M, and PTS-L. An independent *t* test found that LSTA-M, LSTA-L, and PTS-M were significantly different between groups. Cohen *d* calculations resulted in a small effect size for each measurement (Table 1).

**Table 1:** Overall Mean Values for All 6 Measurements^*a*^

	Normative Cohort (n = 150)	Normative Range	Primary ACL Tear Cohort (n = 150)	ACL Tear Range	*P* Value	Effect Size
LSTA-M, deg	10.3 (4.4)	0-30.0	11.4 (4.6)	0-21.0	.**03**	0.24
LSTA-L, deg	9.9 (4.4)	0-28.0	11.1 (4.4)	0-20.0	.**02**	0.27
LSTD-M, mm	24.8 (4.3)	16.2-43.1	24.3 (4.1)	12.1-35.1	.28	0.12
LSTD-L, mm	22.6 (4.1)	13.4-40.0	22.9 (3.9)	10.7-33.3	.45	0.07
PTS-M, deg	9.2 (3.2)	2.0-19.0	10.0 (3.1)	3.0-18.0	.**03**	0.25
PTS-L, deg	9.0 (3.3)	1.0-18.0	9.7 (3.2)	2.0-17.0	.05	0.22

a Data are presented as mean (SD) unless otherwise indicated. Bold *P* values indicate statistical significance. ACL, anterior cruciate ligament; L, lateral; LSTA, lateral supratubercle angle; LSTD, lateral supratubercle distance; M, medial; PTS, posterior tibial slope.

Reliability testing showed that for the normative and primary ACL tear cohorts, there was moderate to excellent agreement between and within raters (Tables 2-4). Intrarater ICCs were all between 0.786 and 0.991 when measuring images obtained in patients with primary ACL tears, which indicates moderate to excellent consistency between measurements made by the same rater over time. Moreover, intrarater ICCs in the normative group were all between 0.784 and 0.997, which indicates moderate to excellent reliability. Interreliability coefficients, as a mean of all judges, were all above 0.844 to 0.993, indicating moderate to excellent consistency between measurements.

**Table 2:** Intrarater ICCs for Each Rater in the Primary ACL Tear Group^*a*^

	Rater 1	Rater 2	Rater 3
LSTA-M	0.822 (0.491-0.940)	0.971 (0.917-0.990)	0.986 (0.957-0.995)
LSTA-L	0.786 (0.381-0.927)	0.934 (0.769-0.979)	0.987 (0.955-0.996)
LSTD-M	0.971 (0.915-0.990)	0.991 (0.975-0.997)	0.960 (0.882-0.986)
LSTD-L	0.930 (0.788-0.976)	0.974 (0.921-0.991)	0.891 (0.676-0.963)
PTS-M	0.972 (0.918-0.990)	0.986 (0.960-0.995)	0.975 (0.927-0.991)
PTS-L	0.844 (0.526-0.948)	0.921 (0.771-0.973)	0.936 (0.813-0.978)

a Data are presented as mean ICC (95% CI). ACL, anterior cruciate ligament; ICC, intraclass correlation coefficient; L, lateral; LSTA, lateral supratubercle angle; LSTD, lateral supratubercle distance; M, medial; PTS, posterior tibial slope.

**Table 3:** Intrarater ICCs for Each Rater in the Normative Group^*a*^

	Rater 1	Rater 2	Rater 3
LSTA-M	0.784 (0.385-0.926)	0.975 (0.807-0.993)	0.949 (0.853-0.983)
LSTA-L	0.798 (0.406-0.932)	0.988 (0.965-0.996)	0.921 (0.762-0.973)
LSTD-M	0.994 (0.983-0.998)	0.997 (0.991-0.999)	0.975 (0.903-0.992)
LSTD-L	0.992 (0.976-0.997)	0.992 (0.977-0.997)	0.951 (0.855-0.984)
PTS-M	0.920 (0.761-0.973)	0.981 (0.924-0.994)	0.928 (0.783-0.976)
PTS-L	0.971 (0.914-0.990)	0.961 (0.888-0.987)	0.965 (0.896-0.988)

a Data are presented as mean ICC (95% CI). ICC, intraclass correlation coefficient; L, lateral; LSTA, lateral supratubercle angle; LSTD, lateral supratubercle distance; M, medial; PTS, posterior tibial slope.

**Table 4:** Interrater ICCs Across All 3 Raters in the Normative and Primary ACL Tear Groups^*a*^

	Normative Cohort	Primary ACL Tear Cohort
LSTA-M	0.904 (0.757-0.966)	0.937 (0.697-0982)
LSTA-L	0.844 (0.576-0.946)	0.915 (0.708-0.973)
LSTD-M	0.993 (0.984-0.998)	0.985 (0.963-0.995)
LSTD-L	0.967 (0.818-0.991)	0.912 (0.737-0.971)
PTS-M	0.975 (0.940-0.991)	0.965 (0.908-0.988)
PTS-L	0.898 (0.762-0.963)	0.905 (0.773-0.965)

a Data are presented as mean ICC (95% CI). ACL, anterior cruciate ligament; ICC, intraclass correlation coefficient; L, lateral; LSTA, lateral supratubercle angle; LSTD, lateral supratubercle distance; M, medial; PTS, posterior tibial slope.

An ROC curve was created to determine the ability of LSTA-L, LSTA-M, and PTS-M to discriminate between the normative and primary ACL tear cohorts. PTS-L, LSTD-L, and LSTD-M were not further analyzed given the lack of significant differences in values between the normative and primary ACL tear cohorts. The AUC was 0.58 (*P* = .02) for LSTA-M, which represents poor discrimination. The cutoff value of LSTA-M was 11° based on mean values, resulting in a sensitivity of 59.3% and specificity of 48.0%. The AUC was 0.59 (*P* = .005) for LSTA-L, which represents poor discrimination. The cutoff value of LSTA-L was 11° based on mean values, resulting in a sensitivity of 57.3% and specificity of 42.7%. The AUC was 0.57 (*P* = .03) for PTS-M, which represents poor discrimination. The cutoff value of PTS-M was 10° based on mean values, resulting in a sensitivity of 52.7% and specificity of 44.7%.

## Discussion

The most novel finding of this study was the establishment of mean values and ranges for LSTA-L, LSTA-M, LSTD-L, and LSTD-M in both normative and primary ACL tear cohorts. Additionally, significant differences in mean LSTA-L, LSTA-M, and PTS-M between the normative and primary ACL tear cohorts were found.

Multiple recent studies have focused on successfully reducing elevated PTS with a slope-reducing osteotomy; however, no consensus has emerged as the preferred location based on the level of the tibial tubercle.^11,21,22,40,41^ To our knowledge, the current literature is void of any normative values for the supratubercle region of the proximal tibia. We propose the LSTA to fill that gap, and analyzed its relationship to PTS on standard lateral knee radiographs. The normative values of LSTA provide angular data, allowing surgeons to determine in patients with elevated PTS if the tibial deformity predominantly occurs in the supratubercle region. These additional data may help us better understand location-specific deformity correction and quantify the changes produced with slope-reducing osteotomies.

LSTA-M and LSTA-L showed small but significant mean measurement differences (LSTA-M: 1.1°, *P* = .03; LSTA-L: 1.2°, *P* = .02) between the normative and primary ACL tear cohorts. Although statistically significant, these differences between cohorts might not be clinically applicable, as a 1° radiographic difference represented a small magnitude of change determined by the effect sizes. Therefore, we hesitate to claim a clinical difference in cohorts with only 1° mean differences. The statistical significance of LSTA-L and LSTA-M between cohorts allowed us to determine cutoff values in relation to PTS. The cutoff values for LSTA-M (11°) and LSTA-L (11°), yielded low sensitivity (59.3% and 57.3%, respectively) and specificity (48.0% and 42.7%, respectively). As such, we cannot confidently state that these threshold values are strong for discriminating between the normative and primary ACL tear cohorts. Comparatively, higher sensitivity and specificity have been reported when assessing the risk of elevated PTS for an ACL tear. When assessing whether PTS was a risk factor for primary ACL reconstruction failure, Ni et al recorded a PTS cutoff value of 17° on full-length lateral radiographs, with a sensitivity of 66.7% and specificity 90.9%.^33^ Duerr et al^9^ went a step further to assess whether PTS was a risk factor for revision ACL graft failure. They found threshold values ≥14° for PTS along the medial plateau and ≥13° for PTS along the lateral plateau on lateral knee radiographs, with calculated sensitivities of 50.00% and 55.3%, respectively, and specificities of 92.1% and 86.8%, respectively. Because of the lower sensitivity and specificity of LSTA, we cannot determine the exact clinical relevance of this measurement. However, we still believe that LSTA may be a useful measurement tool to quantify supratubercle deformity, as now we present mean measurement values and ranges in both cohorts to evaluate this anatomic region.

A major advantage of measuring LSTA is that it is based on proximal anatomic landmarks and is therefore less dependent on the viewable length of the tibial shaft provided on knee radiographs. LSTA and LSTD can be measured on both standard and full-length lateral radiographs without variability in landmarks, contingent on the standard knee radiographs including the proximal aspect of the tibial tubercle. This may improve deformity assessment in practices without available access to full-length lower extremity imaging or that have variability in the tibia length included on standard lateral knee radiographs. This contrasts with PTS assessment that has multiple methods with various results of normal and elevated values, depending on using circles, anterior/posterior cortices, knee versus full tibia radiographs, MRI, and mechanical versus anatomic axis.^‖^

There is debate regarding the effect of a supratubercle slope-reducing osteotomy on patellar biomechanics, as well as whether there is adequate bone stock necessary for this procedure. The concern for the effect of a slope-reducing osteotomy on patellar height stems from the various studies that have shown that high tibial osteotomies (HTOs) for coronal malalignment result in patellar height changes.^1,2,10,23,42^ LaPrade et al^23^ found similar results when assessing knee status after opening-wedge HTO. In 2016, a meta-analysis of 23 studies concluded that patellar height was altered with an opening-wedge HTO, but not after a closing-wedge HTO.^1^ This was evaluated in the slope-reducing osteotomy population by Dejour et al,^7^ who reported a mean decrease in Caton-Deschamps index of 0.07 in a 9-patient cohort at a minimum of 2 years after revision ACL reconstruction with concomitant supratubercle slope-reducing osteotomy. Tollefson et al^43^ also recently demonstrated that patellar height is not significantly altered after a supratubercle anterior closing-wedge slope-reducing osteotomy. We propose that the amount of supratubercle bone available to successfully perform a slope-reducing osteotomy can be determined by preoperative LSTD and may provide a limit to the amount of correction obtainable with the supratubercle technique. To address this concept, Demey et al^8^ evaluated differences in metaphyseal bone stock on standard lateral radiographs between patients with elevated PTS (≥12°) and those with PTS <12°. This study also used anterior tibial metaphyseal height (aHt) to evaluate the sufficiency of metaphyseal bone stock in ACL-deficient knees with the basis resting on measurements of medial PTS on lateral knee films. While aHt and LSTD perform similar measurement functions, we believe measuring the distance along the anterior cortical margin as performed with LSTD evaluates the true anterior bone measurement for available bone stock and potential resultant proximal bone remaining after a supratubercle slope-reducing osteotomy. LSTD, along both the medial and lateral tibial plateaus, showed no significant difference between the 2 cohorts (LSTD-M: 24.8 mm vs 24.3 mm, *P* = .28; LSTD-L: 22.6 mm vs 22.9 mm, *P* = .45). Therefore, no mean difference in proximal supratubercle tibial bone stock between the normative and primary ACL tear cohorts can be inferred from our study. These results agree with those of Demey et al,^8^ finding of no significant difference in anterior metaphyseal height between patients with normal and increased PTS.

### Limitations

Our study has several limitations. The significant difference in mean age between cohorts is a limitation, likely because of the higher risks of ACL tears and subsequent tears in active adolescents.^36^

LSTA measurement was only compared with a single method of PTS calculation using standard lateral knee radiographs. A separate study is needed to compare our findings with PTS measured from full-length lateral radiographs to determine a correlation or if similar findings exist, as well as evaluation over multiple modalities before being generalized for use with CT and MRI. Additionally, although we found small but measurable differences between the normative and primary ACL tear cohorts, the retrospective nature of our study prevented prospective longitudinal risk assessment. These minor differences may also be due to not having a homogeneous ACL tear cohort in these measurements, as with athletes in whom increased shear forces occur during cutting and pivoting. We did not control for contact versus noncontact mechanisms of injury or include only an athletic population. Furthermore, greater differences may have been seen with a comparative control group that does not have knee pain and lives a sedentary lifestyle. Future studies are needed to utilize these established values and ranges to validate these findings before generalizing the results to all patients with ACL injury. We measured LSTD using absolute distance along the anterior cortex instead of creating a normalized ratio, taking into account anterior-to-posterior depth. This causes the measurement to be affected by the overall size of the proximal tibia, with proportional changes not accounted for with absolute measurements. Further research looking at a ratio instead of absolute distance may reflect anatomic differences more accurately.

Future studies will evaluate LSTA as it relates to the decision-making and resultant effect when performing slope-reducing tibial osteotomy, potentially aiding in the selection of either supratubercle or infratubercle osteotomy location. If our values prove to be a reliable indicator in proximal tibial deformity assessment, then more investigation would be needed to examine sufficient bone stock as it relates to LSTA and LSTD, as well as the postoperative changes in these values based on level of correction. We can apply these novel measurements to a myriad of questions to include the effects that osteotomy location has on extensor mechanism biomechanics and secondary deformity creation risk with supratubercle osteotomy. LSTA may also be studied in a longitudinal fashion to confirm if it is truly a more sensitive way to assess proximal tibia morphology and a potential region of tibial deformity as it relates to ACL injury and PTS.

## Conclusion

We have reported novel measurement techniques and normative data for LSTA and LSTD for location-specific measurement of PTS deformity. LSTA-L, LSTA-M, and PTS-M demonstrated small but significant differences between normative and primary ACL tear groups, which may reflect the ability of LSTA to evaluate supratubercle deformity even on standard lateral knee radiographs. These now established values for LSTA and LSTD can be utilized in slope-reducing osteotomy planning by determining if a supratubercle tibial deformity is present. Further studies will build from this groundwork to expand the utilization of these values as they relate to decisions regarding deformity location, augmentation in primary ACL reconstruction, and level of osteotomy correction in revision ACL reconstruction.

## Footnotes

§ References 5, 12, 15, 17, 20, 27, 28, 30, 34, 44.

‖ References 5, 12, 15, 17, 20, 30, 34, 44, 48.
